# A Novel Delay Linear Coupling Logistics Map Model for Color Image Encryption

**DOI:** 10.3390/e20060463

**Published:** 2018-06-14

**Authors:** Shouliang Li, Weikang Ding, Benshun Yin, Tongfeng Zhang, Yide Ma

**Affiliations:** School of Information Science and Engineering, Lanzhou University, Lanzhou 730000, China

**Keywords:** chaos, image encryption, delay and linearly coupled Logistic chaotic map

## Abstract

With the popularity of the Internet, the transmission of images has become more frequent. It is of great significance to study efficient and secure image encryption algorithms. Based on traditional Logistic maps and consideration of delay, we propose a new one-dimensional (1D) delay and linearly coupled Logistic chaotic map (DLCL) in this paper. Time delay is a common phenomenon in various complex systems in nature, and it will greatly change the dynamic characteristics of the system. The map is analyzed in terms of trajectory, Lyapunov exponent (LE) and Permutation entropy (PE). The results show that this map has wide chaotic range, better ergodicity and larger maximum LE in comparison with some existing chaotic maps. A new method of color image encryption is put forward based on DLCL. In proposed encryption algorithm, after various analysis, it has good encryption performance, and the key used for scrambling is related to the original image. It is illustrated by simulation results that the ciphered images have good pseudo randomness through our method. The proposed encryption algorithm has large key space and can effectively resist differential attack and chosen plaintext attack.

## 1. Introduction

With the development of rapid application of computer and internet technology, considerable attention has been paid to the secure transmission of multimedia video information such as texts, images and videos [1]. Therefore, it is becoming more and more important to study an image encryption algorithm with good performance [2,3,4,5]. Chaotic systems are very important systems in nonlinear dynamics. Because of their sensitive dependence on initial conditions and initial values, they are often used in image encryption systems [6,7,8].

Chaos systems generally have one-dimensional and high-dimensional (HD) [9]. However, most of the traditional one-dimensional discrete chaotic maps have the disadvantages of relatively narrow chaos parameter range and small Lyapunov exponents [10]. Therefore, how to design a chaos map with a large Lyapunov exponent, a wide range of chaotic parameters, and the design of efficient and secure image encryption algorithms are currently the focus of research.

The 1D chaotic map usually contains only one variable and a few parameters so that the structure is simple. It is easy to predict [11] the initial conditions and initial values of the mapping, resulting in an image encryption algorithm that is insecure and vulnerable to attack. For example, the encryption algorithms proved to be insecure, which is based on the Logistic map [6,12]. However, although the HD chaotic system has many variables and parameters, the structure is often too complicated and the encryption efficiency is not high. Hua et al. [8] put forward a two-dimensional Sine Logistic modulation map (2D-SLMM) model. In addition, Liu et al. [7] also proposed a system based on two-dimensional sine and an iterative chaotic map with infinite collapse modulation map (2D-SIMM). They all have relatively complex maps in encryption algorithms [13,14]. Their performance evaluation of maps is not ideal, including the fact that the phase diagram and Lyapunov exponent spectrum, complex performance analysis and the implementation of their hardware are relatively complicated. Therefore, a chaotic map with a relatively simple structure and excellent performance is proposed, which can greatly improve the performance of the encryption algorithm, and can make the hardware application relatively simple and inexpensive [15,16,17,18].

In this study, we proposed a novel one-dimensional delay and linearly coupled Logistic chaotic map. It has a relatively simple structure and large enough key space. In the encryption scrambling process of the algorithm, an improvement is proposed over the classical encryption scrambling and diffusion method. In the diffusion process, an improved diffusion strategy is adopted to perform exclusive OR (XOR) operations with the current pixel value not only with the encryption value and diffusion sequence of the previous pixel, but also with the encrypted value of the pixel above the current pixel. The diffusion effect and a good encryption effect are achieved, and the encryption efficiency of the algorithm is improved. In Section 2, we introduced DLCL and analyzed its performance by comparing it with chaotic maps in some other algorithms [7,8,13,14]. We proposed a color image encryption algorithm based on DLCL in Section 3, and we analyzed some of the performance of image encryption algorithms in Section 4. Finally, we provide conclusions in Section 5.

## 2. Delay Linear Coupling Logistics Map

### 2.1. DLCL Model

The structure of delay linear coupling is defined by:

(1) $$X_{n+1}=F\left(X_{n}+aX_{n+1}\right)\;mod1,$$

where *a* is system parameters, and a∈(0,1), When function F(x) is set as the Logistic map, then the DLCL is obtained as:

(2) $$X_{n+1}=u\left(X_{n}+aX_{n+1}\right)\left(1-\left(X_{n}+X_{n+1}\right)\right),$$

where u∈(0,4) is used to enhance its nonlinearity and randomness.

Compared to the 2D-SIMM, 2D-SLMM, the parameter-varying Baker map (PVBM) [13] and the 2D Logistic-adjusted-Sine map (2D-LASM) [14], the structure of DLCL is relatively simple and significantly improves the speed of iteration.

### 2.2. Performance Evaluation of DLCL

#### 2.2.1. Trajectory

**Figure 1:**
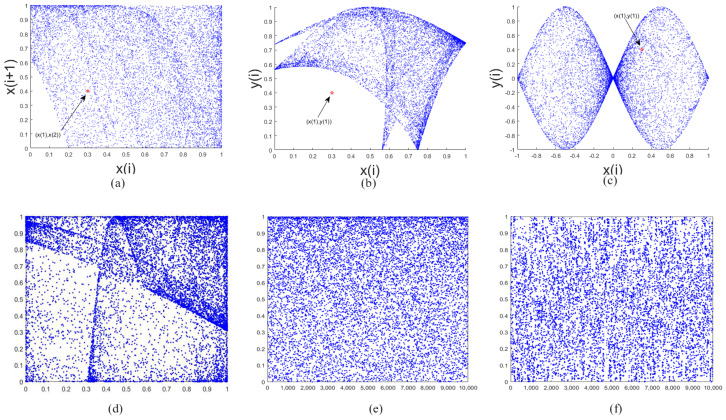
The trajectories of Delay Linear Coupling Logistics (DLCL), two-dimensional Sine Logistic modulation map (2D-SLMM), two-dimensional sine and an iterative chaotic map with infinite collapse modulation map (2D-SIMM), parameter-varying Baker map (PVBM). (**a**) DLCL; (**b**) 2D-SLMM; (**c**) 2D-SIMM; (**d**) 2D-Logistic-adjusted-Sine map (LASM), parameter μ=0.9, initial value $\left(x_{0},y_{0}\right)$ = (0.3, 0.4); (**e**) DLCL, initial value $\left(x_{0},x_{1}\right)$ = (0.6, 0.2), α = 0.8, μ=3.99; (**f**) PVBM, initial value $\left(x_{0},y_{0}\right)$ = (0.2341, 0.0938).

Figure 1 shows the trajectories of DLCL, 2D-SLMM, 2D-SIMM, PVBM and 2D-LASM. For DLCL, 2D-SLMM,2D-SIMM, 2D-LASM, they have the same initial values (0.3, 0.4). We can see that the trajectory of the DLCL is distributed in the region [0,1]×[0,1] from the graph, and, compared with the other three in the same size area, we can see that DLCL has a relatively larger area than 2D-SLMM and 2D-SIMM and DLCL has a more even distribution than 2D-LASM. For DLCL and PVBM, the trajectories of DLCL and PVBM are distributed in the region [0, 1]. This means that DLCL has excellent spatial ergodicity property.

#### 2.2.2. Analysis of Lyapunov Exponent

The sensitive dependence of initial values and initial conditions is the most important feature of chaotic systems. The LE is used to quantitatively characterize the chaotic system, which characterizes the average exponential rate of convergence or divergence between adjacent orbits in a phase space. For discrete systems, the system is in a chaotic state when the LE is greater than zero. The hyperchaotic systems is defined as a chaotic system with several positive LE [19].

**Figure 2:**
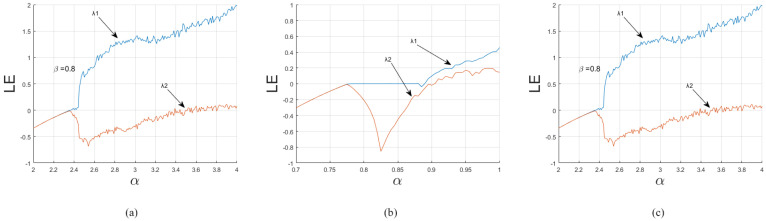
The Lyapunov exponent value of DLCL, 2D-SLMM, and 2D-SIMM. (**a**) DLCL; (**b**) 2D-SLMM; (**c**) 2D-SIMM.

Figure 2 shows the LEs (λ1,λ2) of DLCL, 2D-SLMM and 2D-SIMM. DLCL is chaotic for α∈[2.44,4] and is hyperchaotic for α∈[3.81,4]. 2D-SLMM, when α∈[0.885,1] and α∈[0.905,1], is chaotic and hyperchaotic, respectively. 2D-SLMM, when α∈[0.735,1], is chaotic and, when α∈[0.735,1], is hyperchaotic. Comparison shows that DLCL has a much wider chaotic range, which is six times more than that of other two maps. In addition, DLCL’s LE value is also bigger than that of 2D-SLMM. This means that DLCL is more sensitive to initial values and initial conditions and larger key space can be produced using DLCL.

#### 2.2.3. Analysis of Permutation Entropy

**Figure 3:**
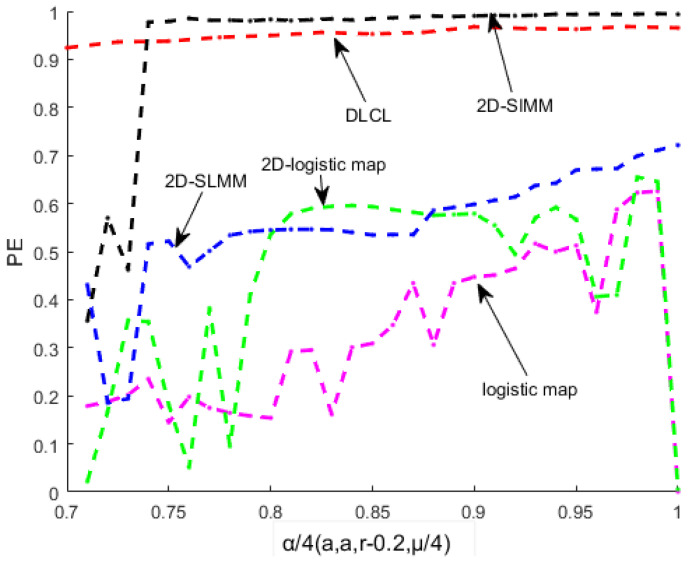
The Permutation entropy of DLCL, 2D-SLMM, 2D-SIMM, 2D-logistic map and logistic map.

Permutation entropy (PE) [20,21] is suitable for measuring the complexity of series of chaos. The larger the value of PE, the more difficult it is to predict the generated chaotic sequence. The PE of DLCL, 2D-SLMM, and the PE of 2D-logistic map, 2D-SIMM and logistic map from Figure 3 can be seen. Obviously, the PE value of DLCL is greater than all maps except 2D-SIMM. The PE value of DLCL and 2D-SIMM are both close to 1 when $\alpha /4\left(a_{0}\right)\in \left[0.74,1\right]$, and DLCL has a wider range than 2D-SIMM. This means that DLCL has better chaotic properties.

#### 2.2.4. Randomness Analysis

National Institute of Standards and Technology (NIST) tests are used to test the randomness of binary sequences generated by hardware or software-based encryption random or pseudo-random number generation programs [22]. A statistical package consisting of fifteen tests. We performed a NIST test on the chaotic sequence map, by setting α=0.01, as long as *p*-value is greater than α, the tests passed:

(3) $$y\left(n\right)=x\left(n\right)\times 10^{k}-floor\left(x\left(n\right)\times 10^{k}\right).$$

In order to improve the randomness of the chaotic sequence, we perturb it in terms of Equation (3). We set *k* = 7, where each sequence is of size $10^{8}$. Tests results show that the sequence we generated passed fifteen tests in Table 1, indicating that the generated sequence has a good randomness.

## 3. Image Encryption Algorithm Based on DLCL

**Figure 4:**
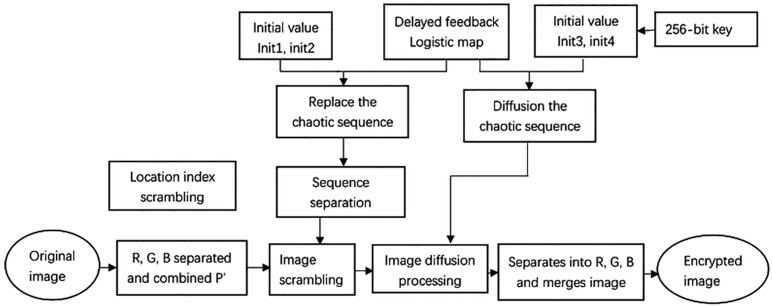
The encryption flowchart of algorithm.

Based on the DLCL model, we propose an image encryption algorithm. Separate R, G, B information from the size of M×N image, and then recombine these three gray-scale images into one image according to certain combination rules and get the image size of M×3N. The chaotic sequence is then used to generate two sets of sorted sequences to perform row and column scrambling on the merged image. In the diffusion process, an improved diffusion strategy is used to XOR the current pixel value with not only the encryption value of the previous pixel and the diffusion sequence, but also XOR with the encryption value of the pixel above the current pixel, so that the diffusion has excellent results. The encryption flowchart of algorithm shows in Figure 4.

### Image Encryption Algorithm

1. Input original color image.
2. Image pre-processing. The color image is separated, and then combined to get a new image according to the Formula (4):
  (4) $$\left\{\begin{array}{c} P\left(:,j\right)=R^{\prime }\left(:,1+\frac{j}{3}\right)\;if\;mod\left(j,3\right)=1,\\ P\left(:,j\right)=R^{\prime }\left(:,1+\frac{j}{3}\right)\;if\;mod\left(j,3\right)=2,\\ P\left(:,j\right)=R^{\prime }\left(:,1+\frac{j}{3}\right)\;if\;mod\left(j,3\right)=0, \end{array}\right.$$
  where j=0,1,2,…,N.
3. The initial value is obtained according to the image $P_{M\times 3N}^{\prime }$, we set init1=0.3 and init2=0.4. A chaotic sequence for permutation is generated. The average value of the pixel $P_{M\times 3N}^{\prime }$ values is averaged and mapped to the range of (0,1) according to the determined transformation formula to obtain the first initial value, the pixel value of the image $P_{M\times 3N}^{\prime }$ is subtracted from the average value of all the pixels, after calculating the variance, the variance is mapped to the range of (0,1) according to the determined transformation formula to obtain the second initial value, and the expression is as follows:
  (5) $$\left\{\begin{array}{c} Init1=(\frac{\sum_{i=1}^{M}\sum_{j=1}^{N}P^{\prime }\left(i,j\right)}{M\times N}+init1)\;mod1,\\ Init2=(\frac{\sum_{i=1}^{M}\sum_{j=1}^{N}{\left(P^{\prime }\left(i,j\right)-\bar{x}\right)}^{2}}{M\times N}+init2)\;mod1, \end{array}\right.$$
  where *M* is the row of input image, *N* is the column of image, and $\bar{x}$ is the average value of all the pixels. By Formula (4), we can get the length of L=M+N series *S*.
4. Given a 256-bit external binary key K, 8-bit as a unit of its block is divided, we can get
  (6) $$K=k^{1},k^{2},k^{3},k^{4},\ldots ,k^{32}.$$
  Generating two initial values of the chaotic sequence according to Formula (8) and substituting the sequence S’ for diffusion:
  (7) $$\left\{\begin{array}{c} init1=(k^{1}⊕k^{2}⊕k^{3}⊕\ldots ⊕k^{32}),\\ init2=(k^{17}⊕k^{18}⊕k^{19}⊕\ldots ⊕k^{32}). \end{array}\right.$$
5. The sequence *S* is used for scrambling and diffusion of the image. First, *S* is divided into two series $S_{1}$ and $S_{2}$ according to Formula (7). Then, $S_{1}$ and $S_{2}$, are used, respectively, to replace the rows and columns of the image $P^{\prime }$:
  (8) $$\left\{\begin{array}{c} S_{1}\left(i\right)=S\left(i\right)\;if\;i\leq M,\\ S_{2}\left(i\right)=S\left(M+i\right)\;if\;i\leq 3N-M. \end{array}\right.$$
  The two subsequences $S_{1}$ and $S_{2}$ obtained in Equation (7) are sorted from small to large. The permutation of the image $P^{\prime }$ is performed according to the subscript array ind1 of the sorted subsequence $S_{1}$. According to the sorted $S_{2}$ subsequence generating the standard array ind2, then column replacement gets a new image $P_{M\times 3N}^{\prime \prime }$;
6. Transform the series $S^{\prime }$ to $S_{M\times N}^{\prime }$ according to two initial values from Formula (7), execute the diffusion to image $P_{M\times 3N}^{\prime \prime }$ according to Formula (9):
  (9) $$\left\{\begin{array}{c} P^{\prime \prime }\left(i,j\right)=P^{\prime \prime }\left(i,j\right)⊕floor\left(S\left(i,j\right)\times 256\right)\;if\;i=1,j=1,\\ P^{\prime \prime }\left(i,j\right)=P^{\prime \prime }\left(i,j\right)⊕P^{\prime \prime }\left(i,j-1\right)⊕floor\left(S\left(i,j\right)\times 256\right)\;if\;i=1,1<j\leq 3N,\\ P^{\prime \prime }\left(i,j\right)=P^{\prime \prime }\left(i,j\right)⊕P^{\prime \prime }\left(i-1,j\right)⊕floor\left(S\left(i,j\right)\times 256\right)\;if\;i=M,j=1,\\ P^{\prime \prime }\left(i,j\right)=P^{\prime \prime }\left(i,j\right)⊕P^{\prime \prime }\left(i-1,j\right)⊕P^{\prime \prime }\left(i,j-1\right)⊕floor\left(S\left(i,j\right)\times 256\right)\;if\;1<i\leq M,1<j\leq 3N, \end{array}\right.$$
  where floor(x) is the smallest integer not greater than x, and ⊕ is the operation that two numbers are bit-XORed by their binary values. $P^{\prime \prime }$ is the encrypted image after diffusion.
7. Let $P^{\prime \prime }$ divide into $R_{M\times N}^{\prime }$, $G_{M\times N}^{\prime }$, $B_{M\times N}^{\prime }$ according to Formula (4). They are then combined for the image $\bar{P_{M\times N}}$ . The image decryption process is the reverse process of the encryption.

## 4. Experimental Results and Analysis of Performance

**Figure 5:**
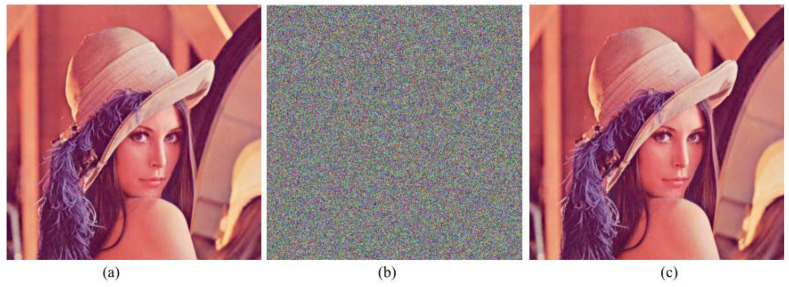
The results of encryption and decryption. (**a**) the original image of Lena; (**b**) encrypted Lena image and (**c**) decrypted Lena image.

**Figure 6:**
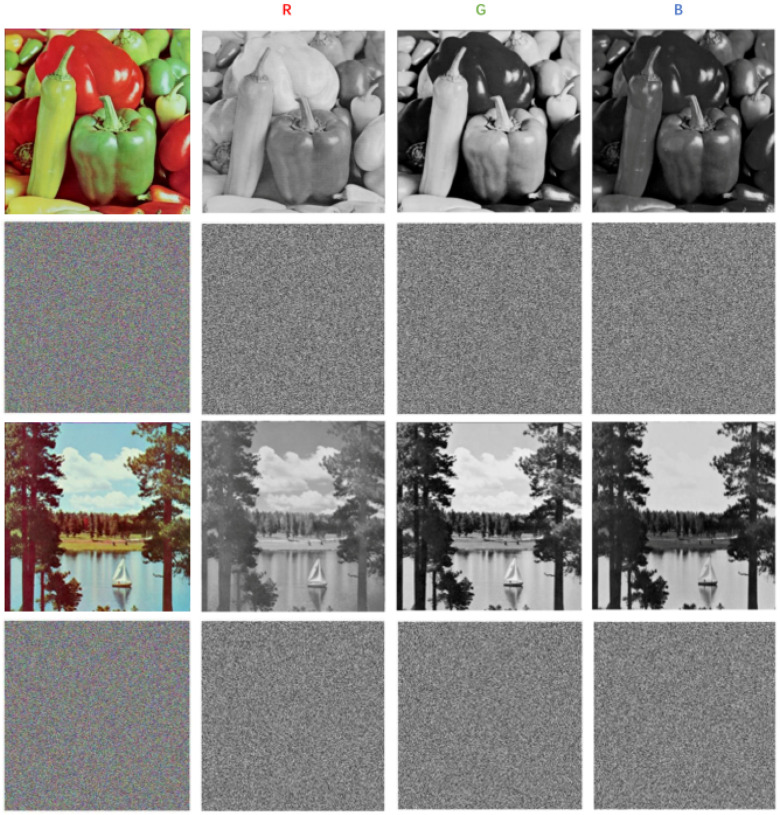
The encryption results of color image and R, G, B components.

We set system parameters *u* as 3.57, α as 0.6, and have one round of encryption of the original image. Figure 5 shows the results before and after the size of 512×512×3 Lena encryption and decryption. Figure 6 shows the the encryption results of R, G, B components, and we can see the encrypted image correctly from the results, in order to show the effectiveness of the algorithm. At the same time, we can see through the histogram and R, G, B image encryption results in Figure 6 that the algorithm can also encrypt the size of M×N gray-scale image effectively.

### 4.1. Secret Key Size Analysis

A good encryption system should have enough large key space to effectively prevent brute force attacks. The cryptographic system key space includes Logistic mapping control parameters, coupling gain and two initial values used to generate chaotic sequences. The proposed encryption algorithm has a 256-bit key, and it has $2^{256}$ of key space. In Table 2, we compare the proposed algorithm’s key space with other algorithms. Therefore, the key space of this paper is sufficient to resist the exhaustive attack and has larger key space.

### 4.2. Secret Key Sensitivity Analysis

**Figure 7:**
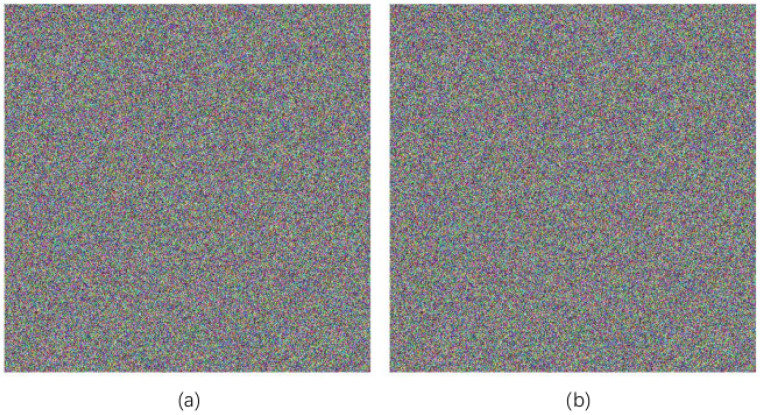
Secret key sensitivity test results. (**a**) α deviates from $10^{-15}$ decrypted images; (**b**) *u* deviates from $10^{-15}$ decrypted images.

Key sensitivity indicates that the key is slightly altered, which can greatly change the decoding result. This image adopts Lena to detect the key sensitivity of algorithms. The key’s offset size is set to $10^{-15}$. The result can be seen in Figure 7, (a) is to make α diverge $10^{-15}$ and (b) is to make *u* diverge $10^{-15}$. It is easy to see in the figure, in the case of $10^{-15}$ deviation from the decryption key, that no meaningful information can be obtained from the decryption result. Therefore, the key sensitivity of the algorithm is strong.

### 4.3. Histogram Analysis

**Figure 8:**
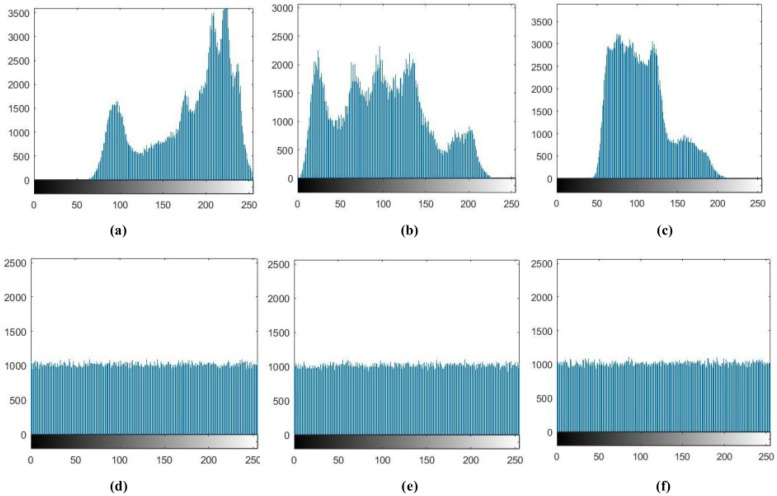
Histograms of the Lena color image and encrypted image. (**a**) histograms of original image R; (**b**) histograms of original image G; (**c**) histograms of original image B; (**d**) histograms of encrypted image R; (**e**) histograms of encrypted image G and (**f**) histograms of encrypted image B.

Image pixel histograms can directly reflect the degree of confusion of image pixels. In the proposed algorithm, each encrypted image pixel is evenly distributed. We can see from the graphs in Figure 8, that the distribution of the plaintext image approaches a diagonal line, indicating that the correlation is strong and the encrypted image is added horizontally, vertically and diagonally. We can also see the distribution of a more uniform direction, indicating that the encrypted image adjacent pixels greatly reduced the correlation between them.

### 4.4. Correlation Analysis

Correlation coefficients between adjacent pixels are another measure of image statistical information. From the image, we select 4000 adjacent pixels in vertical, horizontal and diagonal directions, respectively, and then use Formula (10) to calculate the correlation coefficient:

(10) $$\rho_{xy}=\frac{E\{[x-E(x)][y-E(y)]\}}{\sqrt{D(x)}\sqrt{D(y)}},$$

where $E\left(x\right)=\frac{1}{l}\sum_{i=1}^{l}x_{i}$ is mean, $D\left(x\right)=\frac{1}{l}\sum_{i=1}^{l}{\left[x_{i}-E\left(x\right)\right]}^{2}$ is variance.

The corresponding calculation results tested by the size of 256×256×3 Lena image are shown in Table 3. From this table, after encryption, the correlation coefficient of the image in all three directions is significantly reduced apparently. The correlation of adjacent pixels in each direction of the image before encryption is close to 1, and the result of after encryption is close to 0. This shows that the correlation between adjacent pixels in the encrypted image is greatly reduced and the proposed algorithm has low correlation.

**Figure 9:**
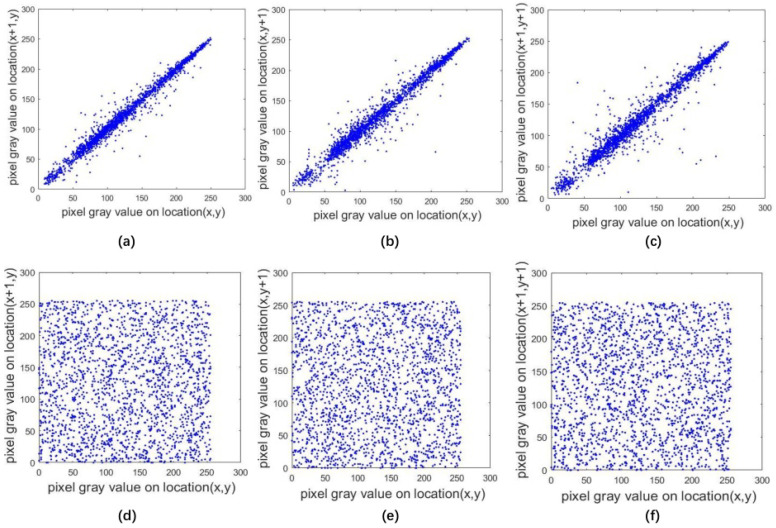
Correlation between plaintext and encrypted images in different directions. (**a**) vertical direction of original image; (**b**) horizontal direction of original image; (**c**) diagonal direction of original image; (**d**) vertical direction of encrypted image; (**e**) horizontal direction of encrypted image; (**f**) diagonal direction of encrypted image.

Figure 9 plots the distribution of the original image’s correlation, indicating that the original image’s correlation is quite strong, and the encrypted image is more evenly distributed in the horizontal, vertical, and diagonal directions.

### 4.5. Analysis of Information Entropy

Information entropy can be used to measure the randomness of image. Let *m* be the source of information in this section, and the formula of information entropy of *m* can be defined as:

(11) $$H\left(m\right)=\sum_{i=0}^{2^{n}-1}P\left(m_{i}\right)log_{2}\frac{1}{P(m_{i})}.$$

$P(m_{i})$ indicates the probability that the symbol appears. For a 256-grayscale image, the ideal value is 8. The closer the information entropy of the encrypted image is to 8, the closer the pixels of the ciphertext image are to the random distribution. We use Lean image with the size of 512×512×3 to calculate the information entropy of the three channels of the encrypted image. From the results in Table 4, it can be seen that the information entropy of the three channels after image encryption is very close to 8. In addition, compared with other algorithms, the information entropy of our proposed algorithm is relatively closer to 8. Therefore, our proposed encryption algorithm can make ciphertext images exhibit good random performance.

### 4.6. Differential Analysis

The more an image encryption system is sensitive to plaintext, the better the ability to resist differential attacks. To describe the sensitivity of the image encryption algorithm to plaintext, we use the number of pixels change rate (NPCR) and unified average changing intensity (UACI) to measure it. The formula can be defined as [31]:

(12) $$\left\{\begin{array}{c} NPCR_{R,G,B}=\sum_{j=1}^{M}\sum_{j=1}^{N}\frac{D(i,j)}{T}\times 100\%,\\ UACI_{R,G,B}=\sum_{j=1}^{M}\sum_{j=1}^{N}\frac{\left|C_{R,G,B}\left(i,j\right)-C_{R,G,B}\prime \left(i,j\right)\right|}{F\times T}\times 100\%, \end{array}\right.$$

(13) $$D\left(i,j\right)=\left\{\begin{array}{c} 0,if\leq C_{R,G,B}\left(i,j\right)=C_{R,G,B}\prime \left(i,j\right),\\ 1,if\leq C_{R,G,B}\left(i,j\right)\neq C_{R,G,B}\prime \left(i,j\right), \end{array}\right.$$

where *T* is number of pixels in total, and *F* is the maximum support pixel values in the image. We can use the NPCR test defined by Equation (14) [32]. If in the NPCR test the encryption algorithm NPCR value is greater than the one-sided hypothesis test under the significance level α defined by Formula (14), it means that the NPCR test passes:

(14) $$N_{\alpha }^{*}=\frac{L-\Phi^{-1}\left(\alpha \right)\sqrt{T/F}}{T+1}.$$

At the same time, we also need to do a UACI test [32], and this test defined by Formulas (15)–(17). It consists of the left value $\mu_{\alpha }^{*-}$ and the right value $\mu_{\alpha }^{*+}$. We choose α = 0.05, and select eight images from the Unversity of Southern California Signal and Image Processing Institute (USC-SIPI) image database. For the size of 256×256 color image, the $N_{0.05}^{*}\geq 99.5693$%, the $\mu_{0.05}^{*-}\geq 33.2834$% and the $\mu_{0.05}^{*+}\leq 33.6447$%. For the size of 512×512 color image, the $N_{0.05}^{*}\geq 99.5893$%, the $\mu_{0.05}^{*-}\geq 33.3730$% and the $\mu_{0.05}^{*+}\leq 33.5541$%. The results in Table 5 show that they all pass NPCR and UACI tests. It can be seen that the image encryption algorithm proposed in this paper is very sensitive to plaintext. Therefore, this algorithm can resist differential attacks well:

(15) $$\mu_{u}=\frac{T+2}{3T+3},$$

(16) $$\sigma_{u}=\frac{\left(T+2\right)\left(T^{2}+2T+3\right)}{18{\left(T+1\right)}^{2}TF},$$

(17) $$\left\{\begin{array}{c} \mu_{\alpha }^{*-}=\mu_{u}-\Phi^{-1}\left(\alpha /2\right)\sigma_{u},\\ \mu_{\alpha }^{*+}=\mu_{u}+\Phi^{-1}\left(\alpha /2\right)\sigma_{u}. \end{array}\right.$$

### 4.7. Encryption Efficiency Analysis

One of the important indicators to measure the performance of image encryption algorithms is encryption efficiency, which has many indicators to measure, such as encryption/decryption time, the encryption throughput (ET) and the number of cycles [33], and they are defined as:

(18) $$ET=\frac{Image_{size}\left(byte\right)}{Encryption_{time}\left(second\right)},$$

(19) $$Number\;of\;cycles\;per\;byte=\frac{CPU_{speed}\left(Hertz\right)}{ET(byte)}.$$

We choose the size of 256×256×3 Lena image. The lab platform is Inter(R) Core(TM) i7-4172MQ CPU@2.30 GHZ with RAM 8.0 GB in MATLAB R2015b (The MathWorks, Inc, Natick, MA, USA) on Windows 8.1 OS (Microsoft, Redmond, WA, USA). Table 6 shows the results that the image encryption algorithms have relatively low complexity and high encryption efficiency. By comparison, we conclude that the proposed encryption algorithm is slower than Refs. [24,34], but quicker than Refs. [23,35]. The algorithm proposed by Murillo et al. [24] is for real-time application. As a result, the encryption time is shorter and the encryption speed is faster and the algorithm of Ref. [34] is for encrypting gray-scale images. The reason why the speed is relatively slow is that our algorithm is complicated in the scrambling and diffusion of images. However, comparing other encryption performances such as information entropy, the algorithm can achieve relative balance in performance.

### 4.8. Robustness Analysis

#### 4.8.1. Quality Metrics Analysis

Quality evaluation of digital images can use the Mean Squared Error (MSE) and Peak Signal-to-Noise Ratio (PSNR) for measurement. They are defined as Equations (20) and (21):

(20) $$MSE=\frac{1}{H\times W}\sum_{i=1}^{H}\sum_{j=1}^{W}{\left(X(i,j)-Y(i,j)\right)}^{2},$$

(21) $$PSNR=10\log_{10}\left(\frac{{\left(2^{n}-1\right)}^{2}}{MSE}\right),$$

where H×W is the size of original image, X(i,j) is the original image and Y(i,j) is the encrypted image. The smaller the MSE value is, the larger the PSNR value is, which means that there is a high degree of similarity between the tested images. By calculation, the MSE between the original image and the decrypted image is 0, and the value of PSNR is Inf. The MSE between the original image and the decrypted image is 30,390, and PSNR is 3.304. The results show that the quality metrics of the tested images is good.

#### 4.8.2. Chosen Plain Image Attack Analysis

**Figure 10:**
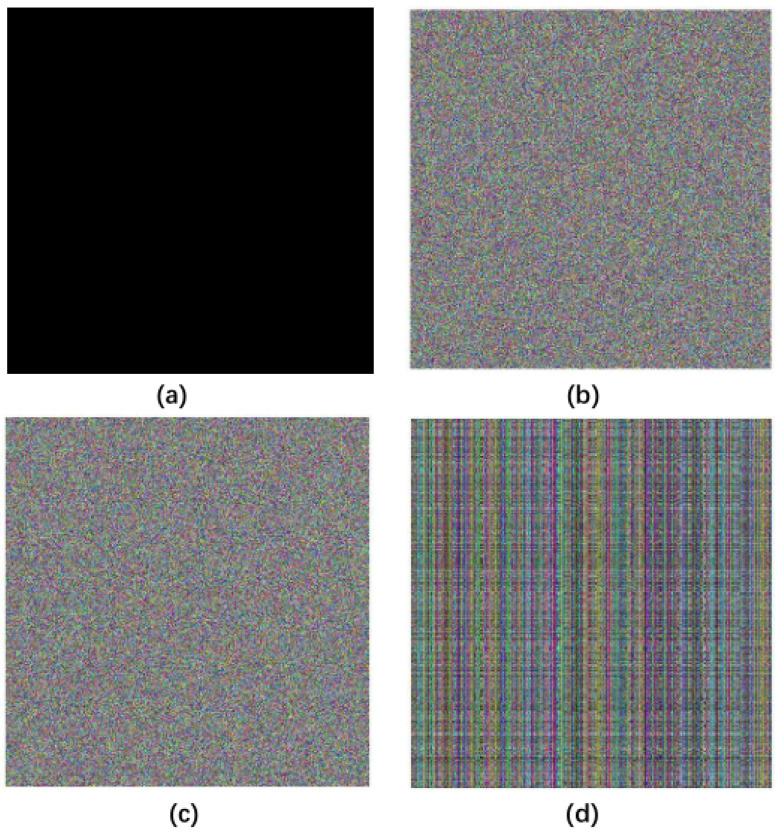
The results of the chosen plain image attack. (**a**) black image; (**b**) encrypted black image; (**c**) encrypted original image; (**d**) decryption of encrypted black image with possible key.

In chosen plain image attack, attackers usually select simple images, such as black images. Because its pixel value is zero, it eliminates the normal image features on the algorithm and the key for encryption. We use the black image for the chosen plain image attack, and the results are shown in Figure 10b. The cryptanalyst uses this information as a possible key and attempts to decrypt other passwords that may be encrypted with the key. Then, we use the possible information to decrypt the original image, and the results show no useful information can be obtained in Figure 10. Therefore, our proposed algorithm can resist the chosen attack.

#### 4.8.3. Occlusion Attack Analysis

**Figure 11:**
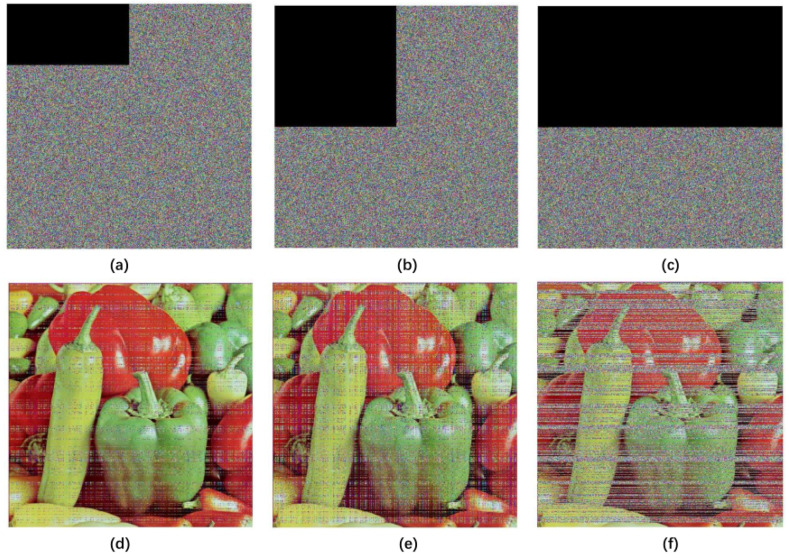
The results of occlusion attack. (**a**) encrypted with 12.5% occlusion; (**b**) encrypted with 25% occlusion; (**c**) encrypted with 50% occlusion; (**d**) decrypted with 12.5% occlusion; (**e**) decrypted with 25% occlusion; (**f**) decrypted with 50% occlusion.

In an occlusion attack, we choose 12.5%, 25%, and 50% of occlusion in an encrypted image. In Figure 11, the attack results are shown. For 12.5% of occlusion, MSE value is 3871.8 and PSNR value is 12.2517. For 25% of occlusion, MSE value is 7727.3 and PSNR value is 9.2505. For 50% of occlusion, MSE value is 15,436 and PSNR value is 6.2456. The results show that the proposed cryptographic algorithm can effectively resist occlusion attack.

#### 4.8.4. Noise Attack Analysis

**Figure 12:**
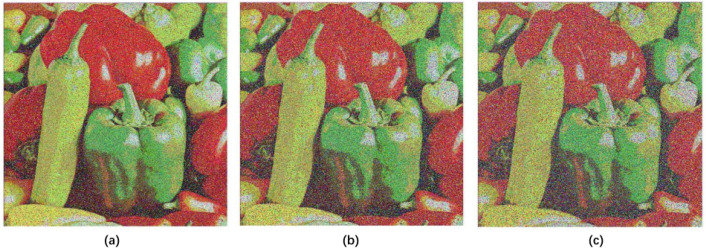
The results of noise attack analysis. (**a**) noise with 10 of intensity; (**b**) noise with 15 of intensity; (**c**) noise with 20 of intensity.

In order to verify the anti-noise performance of the proposed algorithm, Gaussian noise with different intensities was added to the encrypted image. The intensities were 10, 15, and 20, respectively, and they were then decrypted. The results are shown in Figure 12. For 10 of intensity, the MES value is 7900 and PSNR value is 9.1545. For 15 of intensity, the MES value is 10,865 and PSNR value is 7.7704. For 20 of intensity, the MES value is 13,383 and PSNR value is 6.8653. It can be seen that the original image can be basically recovered after the noise image is decrypted. Therefore, the proposed algorithm has a certain anti-noise attack capability.

## 5. Conclusions

We proposed a new one-dimensional delay and linearly coupled Logistic chaotic map in this paper. It has a relatively simple structure, excellent ergodicity property, good sensitivity and better chaotic properties. In the proposed algorithm based on DLCL, through a round of scrambling and diffusion, excellent performance was achieved in many experiments including secret key size analysis, secret key sensitivity analysis, histogram analysis, correlation analysis, information entropy analysis, differential analysis and encryption efficiency analysis. Through the analysis of algorithm performance, this algorithm can resist some common attacks, such as brute force attack, differential attack, statistical attack, chosen plain image attack, and noise attack. Therefore, this algorithm has relatively better encryption performance than other algorithms and is more effective for image encryption applications. In the future, we would improve the construction of chaotic map, reduce the complexity of the algorithm and shorten the encryption time.

## Figures and Tables

**Table 1:** NIST (National Institute of Standards and Technology) test results.

	*p*-Value	Result
ApproximateEntropy	0.909515	SUCCESS
BlockFrequency	0.543991	SUCCESS
CumulativeSums	0.984758	SUCCESS
FFT	0.354010	SUCCESS
Frequency	0.756105	SUCCESS
LinearComplexity	0.174121	SUCCESS
LongestRun	0.097498	SUCCESS
NonOverlappingTemplatel	0.999353	SUCCESS
OverlappingTemplate	0.055895	SUCCESS
RandomExcursion	0.818931	SUCCESS
RandomExcursionsVariant	0.925711	SUCCESS
Rank	0.335464	SUCCESS
Runs	0.531190	SUCCESS
Serial	0.160284	SUCCESS
Universal	0.418957	SUCCESS

**Table 2:** Comparison of key space.

Algorithm	Our Proposed Algorithm	Ref. [23]	Ref. [24]	Ref. [7]	Ref. [25]
Key space	$2^{256}$	$2^{170}$	$2^{128}$	$2^{256}$	$6.5536\times 10^{48}$

**Table 3:** Comparison of the correlation coefficients of two adjacent pixels in Lena with other algorithms.

Color Image	Channels	Original Image			Encrypted Image		
		Horizontal	Vertical	Diagona	Horizontal	Vertical	Diagona
Lean	R	0.9437	0.9710	0.9196	0.0016	−0.0008	0.0020
	G	0.9458	0.9724	0.9234	−0.0001	−0.0039	0.0001
	B	0.8952	0.9437	0.8553	−0.0066	−0.0004	0.0010
Ref. [23]	R	0.9853	0.9753	0.9734	0.0046	−0.0028	0.0013
	G	0.9802	0.9666	0.9630	−0.0009	0.0004	0.0007
	B	0.9558	0.9334	0.9264	−0.0007	−0.0029	−0.0050
Ref. [7]	R	0.9956	0.9780	0.9435	0.0092	0.0053	0.0008
	G	0.9943	0.9711	0.9301	0.0043	−0.0051	0.0095
	B	0.9280	0.9575	0.9093	−0.0037	0.0095	0.0033
Ref. [25]	R	0.9566	0.9812	0.9295	0.0027	−0.0013	0.0039
	G	0.9432	0.9695	0.9199	0.0034	−0.0034	−0.0021
	B	0.9269	0.9586	0.9020	−0.0046	0.0038	0.0013
Ref. [26]	R	0.9400	0.9679	0.8829	0.0024	−0.0009	−0.0147
	G	0.9408	0.9709	0.8646	−0.0056	−0.0036	−0.0295
	B	0.8933	0.9426	0.7451	−0.000664	0.0031	−0.0246

**Table 4:** Information entropy of encrypted images.

Color Image	Encrypted Image			Average of Encrypted Image
	R	G	B	
Lena	7.999218	7.999310	7.999203	7.999243
Ref. [27]	7.997200	7.997200	7.997600	7.997333
Ref. [28]	7.997300	7.997000	7.997100	7.997133
Ref. [7]	7.997500	7.997200	7.997300	7.997333
Ref. [29]	7.997400	7.997100	7.997200	7.997233
Ref. [30]	7.997300	7.996800	7.997200	7.997100
Ref. [24]	7.989300	7.989800	7.989400	7.989500

**Table 5:** Test results of NPCR (number of pixels change rate) and UACI (unified average changing intensity).

Image File	NPCR(%)			UACI(%)			Test Results
	Red	Green	Blue	Red	Green	Blue	
lena (256 × 256 × 3)	99.6323	99.6277	99.5712	33.4913	33.3786	33.4692	Pass
4.1.01.tiff (256 × 256 × 3)	99.6414	99.6124	99.6384	33.6004	33.3232	33.3923	Pass
4.1.02.tiff (256 × 256× 3)	99.5789	99.6368	99.6170	33.3656	33.4348	33.6682	Pass
4.1.03.tiff (256 × 256 × 3)	99.5514	99.6368	99.5941	33.4909	33.4300	33.6542	Pass
4.1.04.tiff (256 × 256 × 3)	99.6475	99.6048	99.6094	33.5038	33.4447	33.4032	Pass
4.2.03.tiff (512 × 512 × 3)	99.5991	99.5846	99.6208	33.4546	33.4330	33.3988	Pass
4.2.05.tiff (512 × 512 × 3)	99.5964	99.6075	99.6212	33.4933	33.4383	33.4691	Pass
4.2.06.tiff (512 × 512 × 3)	99.6056	99.6201	99.5937	33.4249	33.4264	33.4655	Pass

**Table 6:** Time performance analysis and comparison.

	Average Encryption Time (s)	Encryption Throughput (MBps)	Cycles per Byte
Encrypted image	0.35	0.54	4062
Ref. [23]	1.1347	0.165	20,229.45
Ref. [35]	3.6175	0.052	64,189.61
Ref. [34]	0.160	0.39	2445
Ref. [24]	0.1225	1.531	2180.18
